# A Novel Hyperspectral Microscope Imaging Technology for the Evaluation of Physicochemical Properties and Heterogeneity in ‘Xia Hui 6’ Peaches

**DOI:** 10.3390/foods14122099

**Published:** 2025-06-14

**Authors:** Shiyu Song, Zhenjie Wang, Leiqing Pan, Kang Tu

**Affiliations:** 1 College of Food Science and Technology, Nanjing Agricultural University, 666 Bin Jiang Avenue, Nanjing 210095, China; 2 Sanya Institute of Nanjing Agricultural University, Sanya 572024, China

**Keywords:** hyperspectral microscope imaging, peach, physicochemical parameters, heterogeneity

## Abstract

Hyperspectral microscope imaging (HMI) was employed to evaluate the physiochemical properties of and the large intra-variability in individual fruit of ‘Xia Hui 6’ peaches during storage, which gave insights into the heterogeneity of peach fruits at the microscale. The physicochemical characteristics such as firmness (FI), soluble sugar content (SSC), and L* value of peaches showed significant changes, while the microstructure of the tissues broke down. Principal component analysis (PCA) was applied to peach tissues from the sunny side and shady side at different storage stages, which allowed us to clearly visualize the distribution of sugars, water, and pigments at the cellular scale. Single-feature variables were constructed to clarify the correlation between the characteristic bands and physicochemical parameters based on Pearson correlation analysis, with an R^2^ of 0.99 for firmness at 588 nm, 0.98 for titratable acidity (TA) at 432 nm, 0.88 for the L* value at 430 nm and 0.83 for the b* value at 426 nm. This work demonstrated that HMI technology as an accurate and highly effective tool in evaluating the quality of ‘Xia Hui 6’ peaches and targeting, allowing us to visualize the spatial heterogeneity within peach fruit tissues.

## 1. Introduction

Peaches [*Prunus persica* (L.) Batsch], belonging to the Rosaceae family, is widely cultivated around the world [1]. China is the largest peach producer, with a cultivation history of over 4000 years. Attributed to their attractive color, nutrient richness, juicy and soft texture, and fragrant aroma, peaches have become one of the most widely consumed and valued fruits across the globe [2,3]. To a large degree, the quality of peach fruits (color, soluble solid content, total acid, firmness, flavor, storability, etc.) determines consumers’ purchase intention and re-purchase behavior. However, as typical climacteric fruits, peaches undergo quick ripening and senescence during storage, making them highly prone to softening [4], aroma loss [5], and decay [6] after harvest, resulting in a shortened shelf life and serious economic losses [7]. Therefore, developing efficient and accurate techniques for peach fruit quality detection is of great significance for optimizing harvest timing, guiding postharvest handling, and enhancing commercialization.

Currently, various methods are available for evaluating postharvest peach fruit quality. Traditional quality detection tools primarily rely on manual sensory evaluation and destructive physicochemical measurements, such as colorimeters, refractometers, acidometers, texture analyzers, and chromatography. Although reliable, methods using these tools are time-consuming, are destructive, require sample pre-treatment, and lead to difficulties in achieving large-scale detection. Over the past decade, hyperspectral Imaging (HSI) has demonstrated significant potential in the field of agricultural product quality detection due to its combination of the advantages of spectral analysis and image processing [8,9]. HSI enables simultaneous acquisition of spatial information and spectral characteristics from samples, achieving non-destructive detection of chemical compositions and physical properties. HSI has been successfully utilized to evaluate peach quality, such as soluble solid content (SSC) [10], firmness (FI) [11], decay [12], and bruises [13]. Although HSI can allow for the chemical composition assessment of individual fruits at the macroscale, it neglects their unique behavior resulting from tissue-to-tissue or cell-to-cell variations [14]. Moreover, as living organisms composed of cells and tissues, peach fruits experience significant changes at multiple levels that cannot be captured or detected by hyperspectral imaging technology. Hyperspectral microscopic imaging (HMI) is an emerging technology that integrates microscopy and HSI into one system to collect both microscopic spatial and spectral information from the samples [15]. In recent years, HMI has been employed in agricultural product quality inspection to characterize microscopic changes during processing [16], identify foodborne bacteria in meat [17], and evaluate the microstructure and physicochemical properties of infected pears [18].

Fruit heterogeneity refers to the phenomenon that different parts of the same fruit or different individuals of the same species show systematic differences in physicochemical properties, physiological state, and quality characteristics. Peaches are highly heterogeneous fruits with a high degree of internal variability, which is mainly reflected from the stem to the calyx, along the equatorial direction, and from the periphery to the center area. The most obvious intra-variability in individual peach fruits is found between the sunny and shady sides. Due to the difference in the intensity of light and temperature, there are differences in metabolic activities such as pigment synthesis, sugar accumulation, and cell division and differentiation. The variability and heterogeneity of peach fruits may influence the optical propagation properties and interaction behaviors with incident light [19], resulting in different optical responses. During peach storage, various metabolic activities lead to alterations in both chemical composition and microstructure, which motivated our application of HMI technology to characterize peach fruit heterogeneity at the microscopic level and monitor postharvest quality attributes. To the best of our knowledge, it has been largely absent even very recently from efforts to understand the heterogeneity of peach fruits using HMI.

In this study, the differences in spectral characteristics and quality parameters of the sunny and shady sides during storage were systematically analyzed by HMI combined with chemometric methods. The objectives of this study are to (1) compare the spectral response characteristics and physicochemical indicators of the sunny and shady sides and (2) visualize the spatial distribution of biochemical components at the tissue level.

## 2. Materials and Methods

### 2.1. Sample Preparation

‘Xia Hui 6’ peaches of the same maturity were harvested from a local orchard in Nanjing, Jiangsu Province, China, in 2024 on 8 July (after 110 days of blooming). They were transferred to a laboratory immediately for further experiments. To minimize the influence of sample differences on the results, ‘Xia Hui 6’ peaches of similar appearance and size, and without any visual defects, bruises, and visible diseases, were carefully selected. To simulate real shelf storage conditions, a total of 60 peaches (6 storage periods × 10 samples at each storage period) with a mean equatorial diameter of 78.63 ± 3.20 mm and weight of 237.61 ± 19.35 g were stored at 20 ± 1 °C, 65% RH, for seven days. For each sampling time (Day 0, 1, 2, 3, 5, 7), 10 samples were randomly taken for spectral detection and physiochemical analysis. There were two sampling points (marked regions)—the sunny and shady sides—around the equator of each peach for analysis. The fruit firmness, SSC, and total acid of each peach sample were determined. For each experiment, the HMI spectra of 10 peaches were initially acquired at the marked region; this was followed by the determination of physiochemical indicators.

### 2.2. Hyperspectral Microscope Imaging Acquisition

Hyperspectral data of ‘Xia Hui 6’ peaches were acquired using the Vis-NIR HMI system in transmission mode. The system comprised a hyperspectral imaging spectrometer (4250, HinaLea Imaging, Emeryville, CA, USA), an upright microscope with a 20× objective lens (BMC303-IPL, Phenix Optics Co., Shangrao, China), and a computer equipped with TruScope software (Ver 1.1.6, HinaLea Imaging, Emeryville, CA, USA). A total of 299 bands in the range of 400–1000 nm with a spectral resolution of 2 nm in the transmittance profiles were retained. To ensure the stability of the HMI system and the uniformity of lighting, the system was warmed up for 20 min. Then, the HMI system was calibrated in black and white using a quartz glass slide. A three-dimensional data hypercube of 968 × 608 × 299 was finally obtained.

**Figure 1:**
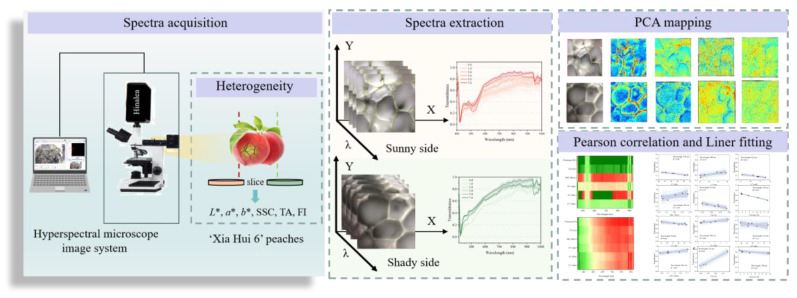
Flowchart demonstrating the methodology used for spectra acquisition and data analysis.

After removing approximately 5 mm of skin from the surface of the specified regions, pulp slices with a thickness of 0.5 mm were obtained using a razor blade. Each slice was transferred onto a quartz glass slide and placed on the microscope stage for HMI acquisition and analysis. To acquire clear images, the parameters of the HMI system were set after pretesting as follows: the light source intensity was 35 W, while the camera was in automatic exposure mode. A total of 120 data cubes were obtained from 60 peaches (10 peaches × 2 slices × 6 storage times) in total. Figure 1 presents a flowchart of this study.

### 2.3. HMI Data Extraction

It is of great importance to select a proper region of interest (ROI), as it can reflect the actual characteristics of samples. In this research, a square area (60 × 60 pixels) centered on the sunny and shady sides was defined as the region of interest. The calculation of mean spectra values of all pixels in the ROI was performed by extracting the spectral information using TruScope software.

### 2.4. Determination of Physical and Chemical Indicators

#### 2.4.1. Determination of Color and Firmness

The color of ‘Xia Hui 6’ peaches was measured using a CR-10 digital portable colorimeter (Minolta Co., Tokyo, Japan). The L*, a*, and b* values were measured three times at the sunny and shady sides, and the average value was utilized for subsequent analyses.

Firmness measurements of the peach flesh tissues were conducted on the sunny and shady sides, which are the same areas used for collecting the HMI spectra. Puncture tests were performed in peach flesh using a digital TMS-Pro texture analyzer (FTC, Arlington, VA, USA) equipped with a 6 mm cylindrical probe. The testing speed was set at 2.0 mm/s, the trigger force was 0.3N, and the penetration depth was 8.0 mm. The results were expressed in Newtons (N).

#### 2.4.2. Determination of SSC and Titratable Acidity (TA)

SSC was determined using a PAL-1 digital refractometer (ATAGO, Tokyo, Japan) based on the juice that was acquired by squeezing and filtering the peach flesh. Similarly, TA was determined by a portable acidometer (PAL-Easy ACID, ATAGO, Japan) using the same juice [20]. The results were expressed in percentage (%).

### 2.5. Data Analysis

Principal component analysis (PCA) is one of the most widely used chemometric methods for data reduction and shows great advantages in processing high-dimensional spectral data [21]. As a classical statistical feature-extraction chemometric tool, it compresses original variables into a new coordinate space known as principal components (PCs) based on the method of linear projection to recognize and highlight characteristics and their correlation to the physiochemical properties of the sample [22,23]. In this research, PCA was performed to highlight the differences in the sunny and shady sides and in two-dimensional PC space. The selection of principal components (PCs) as predictive variables was determined by their score distributions, followed by conversion to false-color image representations. Spectral fingerprint regions and molecular constituents were then characterized through loading plot analysis [24] to explain the difference between the two sides and changes during storage.

### 2.6. Statistical Analysis and Software

One-way analysis of variance (ANOVA) and Duncan’s test (on physicochemical parameters: L*, a*, and b* values, SSC, and TA) were conducted on a total of 60 apples using IBM SPSS 27 statistical software (SPSS Inc., Chicago, IL, USA). The experimental data were presented as mean values of the recorded data, along with their corresponding standard deviations. Pearson correlation analysis and PCA were conducted and false-color images were captured using MATLAB R2023a (MathWorks. Inc., Natick, MA, USA). Correlation coefficients between all quality parameters, a linear regression model, and all the graphs were generated using Origin 2024b (Origin Lab Corporation, Northampton, MA, USA).

## 3. Results

### 3.1. Physicochemical Characterization Analysis

**Figure 2:**
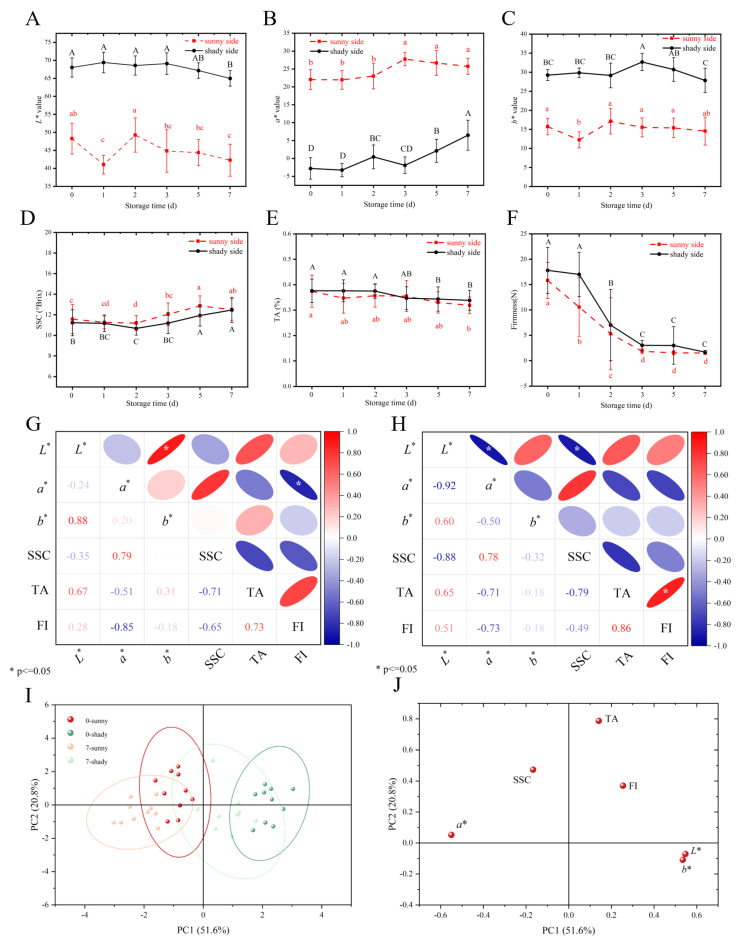
Changes in the L* value (**A**), a* value (**B**), b* value (**C**), soluble solid content (**D**), total acid (**E**), and firmness (**F**) during storage of ‘Xia Hui 6’ peaches. Pearson correlation analysis for physicochemical parameters on the sunny and shady sides (**G**,**H**). The results of the PCA analysis of physicochemical indicators on 0 d and 7 d on the sunny and shady sides (**I**,**J**). In (**A**–**F**), different lowercase letters indicate significant differences (*p* < 0.05) in the sunny side and different uppercase letters indicate significant differences (*p* < 0.05) in the shady side during different storage times.

The changes in physicochemical indicators on the sunny and shady sides during storage are shown in Figure 2.

Color serves as the most direct indicator for evaluating peach postharvest quality. In this study, L* (lightness) values on the sunny and shady sides exhibited an overall decreasing trend; a* values exhibited an overall increasing trend (becoming red), which was related to the ripening during storage; and the b* (indicating yellowness) value expressed no significant change. Flesh firmness also serves as a crucial indicator for evaluating fruit quality, as a decrease in firmness is typically associated with fruit ripening and aging, serving as a key metric for monitoring developmental changes in horticultural products. As ‘Xia Hui 6’ peaches are typical hard-melting fruits, their firmness decreased significantly and rapidly in the early stages of storage (0–3 d), from 15.80 ± 3.56 N to 1.49 ± 0.29 N and from 17.80 ± 4.53 N to 1.68 ± 0.33 N, respectively, but there was no significant change during 4–7 d. The softening of peach flesh could be attributed to increased respiratory metabolism, decreased intercellular adhesion, and changes in cell wall polysaccharides [25].

SSC and TA are typical components responsible for the flavor and taste of peach fruits. In this study, the SSC on both the sunny and shady sides exhibited an overall increasing trend throughout the entire storage period, from 11.58 ± 1.41% to 12.51 ± 1.04% and from 11.23 ± 1.26% to 12.46 ± 1.22%, respectively. At each storage time, the SSC of the sunny side was consistently higher than that of the shady side, with statistically significant differences observed particularly on 3 d and 5 d. The TA contents on the two sides exhibited an overall decreasing trend throughout storage, with no significant difference observed between the two groups until 7 d of storage. This change can be attributed to malate and citrate metabolism. As the main organic acids in peaches, malate and citrate are consumed as respiratory substrates through the tricarboxylate citrate cycle to provide energy for fruit metabolism [26].

Pearson’s analysis was employed to examine the relationships between the physicochemical parameters of ‘Xia Hui 6’ peaches, and the results are displayed in Figure 2G,H. On both the sides, SSC was strongly correlated with values having correlation coefficients (r) of 0.79 and 0.78 (*p* < 0.05), respectively. This may be attributed to the synergistic regulation of anthocyanin synthesis and sugar accumulation in peach fruits. Notably, firmness exhibited strong correlations with multiple physicochemical parameters, demonstrating its critical role in monitoring peach fruit changes during storage.

It can be observed from Figure 2I that the four stages can be grouped. As shown in Figure 2J, the cumulative contribution of PC1 and PC2 reaches 72.4%, which represent the characteristic differences in the original variables. The physical and chemical indicators of the peaches at 0 d and 7 d showed a regular distribution with PC1, where the samples of the sunny group were distributed in the second and third quadrants and the samples of the shady group were distributed in the first and fourth quadrants. From the loadings plot, the L* value, b* value, and TA were the key indicators for distinguishing between the sunny and shady sides.

Accordingly, the above results demonstrate that the significant variations in FI, SSC, and TA of ‘Xia Hui 6’ peaches exhibited significant dynamic changes during storage.

### 3.2. Spectral Characteristics of ‘Xia Hui 6’ Peaches

**Figure 3:**
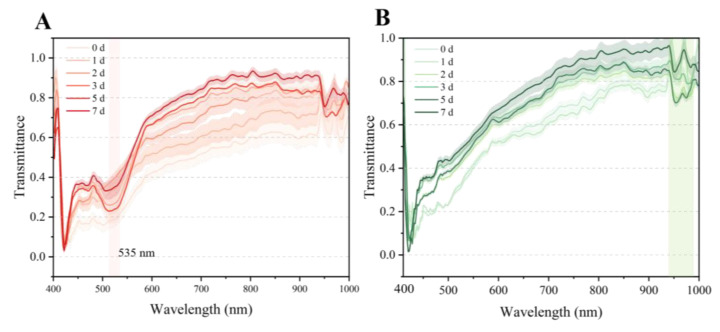
The original mean transmittance spectra of ‘Xia Hui 6’peaches during storage: sunny side (**A**), shady side (**B**).

The average transmittance spectra with standard deviations for the different sides of ‘Xia Hui 6’ peaches in the range of 400–1000 nm are expressed in Figure 3. It can be observed that all samples at different storage periods maintained comparable vibration patterns but still exhibited minor variations, which can be attributed to changes in the chemical composition and tissue structure of the peach fruits. The spectral waveforms of the sunny and shady sides are similar but varied especially in the visible region (480–550 nm), primarily due to pigment content [27]. Particularly, for the sunny side, a distinct transmission trough around 535 nm can be observed during late storage, primarily due to anthocyanin accumulation in the flesh during peach ripening, which enhanced light absorption and consequently reduced transmittance [28]. The shady side generally showed higher spectral transmittance than the shady side during storage at 0–3 d wherein the major wavelength ranges were 450–600 nm and 900–980 nm, but beyond these ranges, the transmittance values increased. This can be attributed to flesh breakdown and firmness loss [29], as previous studies have shown that loosening of tissue structure can lead to increased light scattering [30].

The spectra exhibited distinct troughs at wavelengths associated with significant absorption (480 nm, 950 nm, and 980 nm), which are related to beta-carotenoid absorption [31]. The spectral bands at 950 nm and 980 nm correspond to characteristic vibrational modes of O-H stretching in water molecules and C-H bending in carbohydrate compounds, respectively [32]. The transmittance of the sunny side showed relatively lower values than the shady side, which was consistent with the lower firmness of the sunny side compared to the shady side. Moreover, the spectral changes in the later stage of storage were not as significant as changes in the early stage, which was consistent with the physicochemical changes.

### 3.3. PCA Mapping

**Figure 4:**
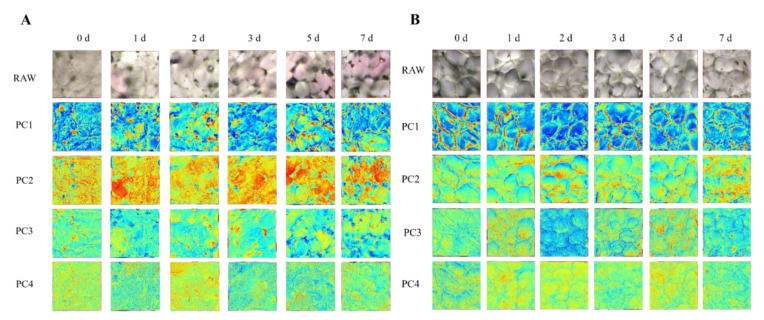
The first four PCs obtained from the HMI spectral region (430–980 nm) during storage: sunny side (**A**), shady side (**B**).

To enhance the visualization of physicochemical changes and differences in the two sides in peach tissues during storage, PCA was conducted on the hyperspectral images of samples. The photographed images based on the first four PC scores are displayed in Figure 4. The PC1 images clearly show the cell walls in the peaches, explaining 92.52% and 91.06% of the variance in the sunny and shady sides, respectively. The PC2 image mainly shows the distribution of cell contents. It can be seen that, due to differences in pigment content, the sunny and shady sides exhibit significant differences. In contrast, the PC3 and PC4 images exhibit more noise due to the lower variance explained. Additionally, as the storage time increases, the cells in both areas show blurred cell boundaries, mainly due to water loss and cell loosening during storage. By comparing the PCA images of the sunny and shady sides, it can be observed that microscopic differences exist primarily in terms of pigment contents and the tightness of cell bonding. The latter can explain why the firmness of the shady side is higher than that of the sunny side, which is attributed to the spatial heterogeneity of cell structure and composition. The PCA demonstrates the differences in the distribution of chemical substances in different parts of the peach fruits, allowing us to visualize the distribution of chemical components (pigment, sugar, water, etc.) in fruit tissue during storage.

### 3.4. Pearson Correlation and Linear Fitting

**Figure 5:**
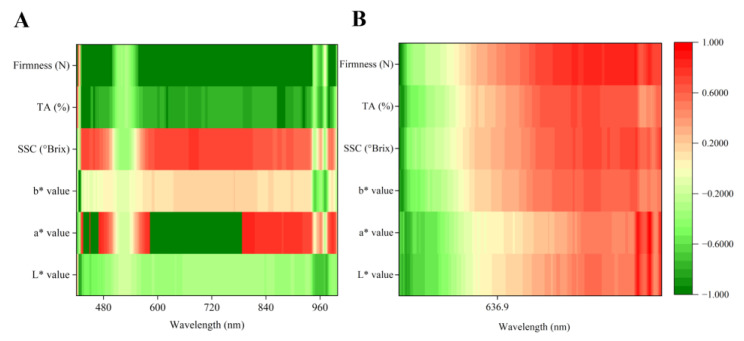
Pearson correlation analysis of two different regions’ spectral data with physicochemical parameters: sunny side (**A**), shady side (**B**).

Pearson correlation analysis was conducted using the two sides’ spectra in the 420–1000 nm range and the physicochemical parameters (L* value, a* value, b* value, SSC, and TA) during storage. The correlation coefficients (r) and wavelengths are shown in Figure 5. Among all the physicochemical parameters, firmness had the strongest correlation on the sunny side, with r = −0.994 (*p* < 0.01) at 588 nm, followed by the L* value (r = −0.940, *p* < 0.01) on the same side at 420 nm. In addition, strong correlations were also identified for the b* value and TA, with r = −0.909 for the L* value at 426 nm and r = −0.882 for TA at 432 nm. The correlation coefficient values and corresponding wavelengths for each physicochemical parameter of the fruit sides are presented in Table 1. Notably, significant differences were observed in correlation coefficients and characteristic wavelengths for the same physicochemical parameter on the sunny and the shady sides, with generally stronger correlations on the sunny side. The characteristic wavelengths were concentrated at 420–440 nm on the sunny side but predominantly at 970–980 nm on the shady side. This further demonstrates the pronounced heterogeneity within peach fruit tissues.

**Figure 6:**
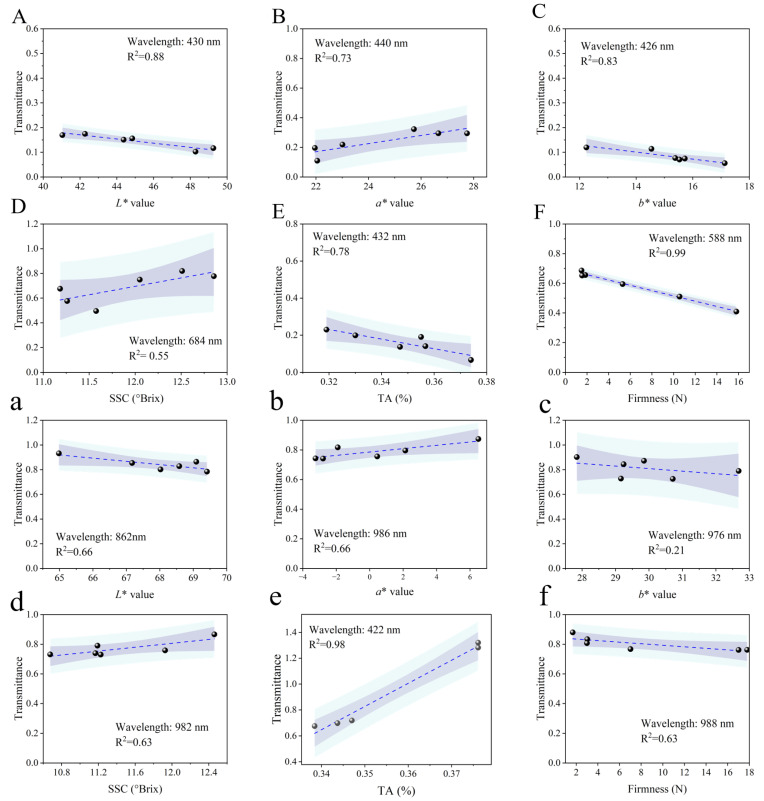
Linear fitting results for physicochemical parameters based on characteristic wavelengths: sunny side (**A**–**F**), shady side (**a**–**f**).

Linear fitting was used to clarify the correlation between the characteristic bands and physicochemical parameters for the ‘Xia Hui 6’ peaches. Figure 6 presents the linear fitting results, and the purple shading and blue shading represent 95% confidence intervals and the prediction band, respectively. A strong linear relationship was observed for firmness, with an R^2^ of 0.99 at 588 nm on the sunny side, whereas TA showed a highly linear relationship for the shady side data, with an R^2^ of 0.98 at 432 nm. Accordingly, the implementation of linear fitting allowed us to successfully extract feature wavelengths, enabling accurate quantification of quality attributes, especially for firmness and L* values.

## 4. Conclusions and Discussion

In this study, firmness changed significantly, from 15.80 ± 3.56 N to 1.49 ± 0.29 N and 17.80 ± 4.53 N to 1.68 ± 0.33 N, respectively, while SSC and TA observed significant changes at the end of storage. During the later stage of storage, significant differences were observed between the sunny and shady sides in SSC, TA, and firmness. In addition, the spectral patterns from the sunny and shady sides present similar vibrational profiles, but with slight differences in spectral intensity, especially in the range of 480–550 nm. Moreover, PCA analysis was conducted on the hyperspectral images to explore the possibility of chemical composition and cell structure in the peach flesh at the cellular scale. Linear fitting was used to establish mathematical relationships between characteristic wavelengths and physicochemical parameters, with an R^2^ of 0.99 for firmness at 588 nm, 0.98 for TA at 432 nm, 0.88 for the L* value at 430 nm, and 0.83 for the b* value at 426 nm. In conclusion, HMI technology proves the effectiveness of hyperspectral imaging features and spectral variables in evaluating the postharvest quality of peaches. This study also demonstrated high variability in spectral transmittance in different regions in a single peach. The observed differences in physicochemical properties between the sunny and shady sides fundamentally stem from spatial heterogeneity in microscopic cellular structure and chemical constituent distribution. In our study, the spectral data from the sunny side showed better linear fitting performance for physicochemical properties compared to the shaded side. Datasets with sufficient variability can help us build robust predictive models, and for highly heterogeneous samples, more data analysis methods can be used to improve model performance. However, for highly heterogeneous fruits, data from a single side cannot fully represent the whole fruit. Therefore, for further research, collecting a sufficiently diverse dataset that encompasses adequate variability is crucial for building more robust prediction models.

## Figures and Tables

**Table 1:** Pearson correlation results for different regions and physicochemical parameters.

Parameter	Range	Side	Wavelength (nm)	Correlation Coefficient
L* value	41.04–49.25	Sunny	430	−0.940
	64.98–69.41	Shady	862	−0.810
a* value	21.96–27.75	Sunny	440	0.856
	−3.24–6.5	Shady	986	0.810
b* value	12.24–17.13	Sunny	426	−0.909
	27.84–32.68	Shady	976	−0.455
SSC (°Brix)	11.26–12.86	Sunny	684	0.742
	10.68–12.46	Shady	982	0.797
TA (%)	0.33–0.37	Sunny	432	−0.882
	0.34–0.38	Shady	422	0.807
Firmness (N)	1.49–155.80	Sunny	588	−0.994
	1.68–17.80	Shady	988	−0.797

## Data Availability

Data are contained within this article.
